# Quantitative evaluation of microtwins and antiphase defects in GaP/Si nanolayers for a III–V photonics platform on silicon using a laboratory X-ray diffraction setup^1^

**DOI:** 10.1107/S1600576715009954

**Published:** 2015-05-31

**Authors:** Yan Ping Wang, Antoine Letoublon, Tra Nguyen Thanh, Mounib Bahri, Ludovic Largeau, Gilles Patriarche, Charles Cornet, Nicolas Bertru, Alain Le Corre, Olivier Durand

**Affiliations:** a UMR FOTON, CNRS, INSA Rennes, Rennes, F35708, France; b Universite Grenoble Alpes, Institu NEEL, Grenoble, F-38000, France; c CNRS, Institut NEEL, Grenoble, F-38042, France; d Laboratoire de Photonique et Nanostructures, CNRS UPR 20, Route de Nozay, Marcoussis, 91460, France

**Keywords:** microtwins, antiphase defects, GaP/Si nanolayers, semiconductors, laboratory X-ray diffraction

## Abstract

This study is carried out in the context of III–V semiconductor monolithic integration on silicon for optoelectronic device applications. X-ray diffraction is combined with atomic force microscopy and scanning transmission electron microscopy for structural characterization of GaP nanolayers grown on Si. GaP has been chosen as the interfacial layer, owing to its low lattice mismatch with Si. But, microtwins and antiphase boundaries are still difficult to avoid in this system. Absolute quantification of the microtwin volume fraction is used for optimization of the growth procedure in order to eliminate these defects. Lateral correlation lengths associated with mean antiphase boundary distances are then evaluated. Finally, optimized growth conditions lead to the annihilation of antiphase domains within the first 10 nm.

## Abstract 2

A laboratory X-ray diffraction setup is reported, which allows quantitative characterization of the microtwin and antiphase domain densities in epitaxial GaP/Si thin layers.

## 1. Introduction   

The heterogeneous epitaxy of III–V compounds on Si substrates has been widely studied for decades in the context of low-cost monolithic integration of III–V photonics and photovoltaics on silicon. However, the lattice mismatch between most III–V semiconductors and Si leads to threading dislocations (Fang *et al.*, 1990 ▶; Bartenlian *et al.*, 1992 ▶). The coherent growth of quasi-lattice-matched (0.36% at room temperature) GaP on Si has therefore been proposed, to provide an alternative route for dislocation-free pseudo-substrates which permits the subsequent over growth of III–V semiconductors (Yonezu *et al.*, 2008 ▶; Guo *et al.*, 2009 ▶). However, planar defects such as antiphase domains (APDs) and microtwins (MTs) generated at the GaP/Si interface are difficult to avoid and detrimental to the optoelectronic properties of devices. Therefore, it remains one of the key issues to develop a fast and reliable structural analysis strategy to detect and lower the defect densities.

Transmission electron microscopy (TEM) has been widely employed for *ex situ* analysis of APDs and other typical defects (Németh *et al.*, 2008*a* ▶,*b* ▶). However, this requires sample preparation and more work in TEM analysis itself, for reliable quantification of the defects. In this paper, we report several evaluation methods of MT and APD defects in GaP nanolayers deposited on Si(001) 6°-off substrates, based on X-ray diffraction (XRD). The pole figure method is used for simple visualization of scattered signals of MT that correspond to the relative MT density level. ‘Rocking curves’ are employed for a more reliable absolute quantification of the MT volume fraction in the total layer. The mean antiphase boundary (APB) distances are investigated by high-resolution reciprocal space mapping around the Bragg positions of GaP 002 and 006. Finally, complementary scanning transmission electron microscopy (STEM) techniques are used to give a complete structural analysis.

## 2. Experimental method   

### 2.1. Sample growth   

The samples of this study were grown under a molecular beam epitaxy (MBE) system on (001)-oriented Si 6°-off substrates toward the [110] direction to favour the APD density limitation (Kroemer, 1987 ▶; Sieg *et al.*, 1998 ▶; Volz *et al.*, 2011 ▶). The substrates were prepared by an HF-last cleaning process consisting of a diluted HF dip followed by exposure under UV/O_3_ environment and a last diluted HF dip (Quinci *et al.*, 2013 ▶). A first set of three 45 nm GaP/Si samples is presented for MT measurements and growth procedure optimization. Samples S1 and S2 were grown by using the conventional MBE mode with a two-step procedure: a 10 nm-thin layer grown at 623 K, followed by a 35 nm-thin layer grown at 853 K, with a P prelayer for S1 and a Ga prelayer for S2. Sample S3 was grown at 623 K using a nonconventional MBE technique called migration enhanced epitaxy (MEE), which consists of alternated growth of Ga and P atomic layers, here with Ga as prelayer. This technique allows a two-dimensional growth mode even at relatively low growth temperature (Takagi *et al.*, 1998 ▶). APD analyses were performed on two other 45 nm GaP/Si samples: S4 and S5. Both were grown using the same two-step procedure, with a first 10 nm-thin MEE GaP layer grown at 623 K, followed by a continuous 35 nm-thin MBE GaP layer grown at 773 K, except for a slight difference in the Ga content per MEE cycle at the early stage of growth for S5. Sample S6 was grown explicitly for STEM study, by using the same growth procedure as S5 for the first 10 nm-thin layer, followed by successive MBE GaP layers with increasing growth temperatures of 773, 808, 838 and 873 K. Each MBE layer was separated by one AlGaP marker.

### 2.2. X-ray diffraction experiments   

X-ray diffraction was performed on a four-circle Brucker D8 diffractometer (horizontal scattering plane geometry) using two different modes of this instrument: the standard low-resolution mode and a high-resolution mode. This diffractometer is equipped with a one-dimensional Göbel multilayer mirror placed on the linear focus window of a standard sealed tube as primary optics. The feeding power is set at 40 kV/40 mA. The detector is a LynxEye one-dimensional position-sensitive detector (PSD). It is used in either PSD or point detector mode. This PSD is positioned at 300 mm from the goniometer centre and presents 180 channels, making a maximum of 13.5 mm (2.6°) in the horizontal direction.

#### 2.2.1. Low-resolution mode   

The beam is limited in height and width to about 2 × 2 mm by a cross-slit system to produce a quasi-point beam. The LynxEye detector is used in point-detector mode, with an 8 mm width aperture in the horizontal direction and the full width aperture (about 15 mm) in the vertical direction, to ensure a full capture of the MT scattered signal. An Ni filter is placed before the detector to reduce the *K*β pollution and to select the mean Cu *K*α rays with a wavelength of 0.154184 nm. A 2.5° Soller slit is also placed here for background reduction, after checking the absence of side effects on quantitative measurements.

#### 2.2.2. High-resolution mode   

A four-bounce Ge(022) asymmetric monochromator (Bartels) is used to reduce the divergence of the X-ray beam down to 29′′ and also to select the *K*α_1_ rays with a wavelength of 0.154056 nm. The full height of the beam is employed (instead of 2 mm in low-resolution mode) and the Ni filter is removed. Reciprocal space maps (RSMs) are recorded by the LynxEye detector working in PSD mode.

### 2.3. Other structural analysis experiments   

Atomic force microscopy (AFM) measurements (5 × 5 µm) were performed on a 2007 Veeco Innova system in contact mode.

Scanning transmission electron microscopy bright-field (STEM-BF) imaging was performed using a TEM/STEM Cs-corrected JEOL 2200 FS operated at 200 kV. The effective STEM spot size resolution is 0.1 nm.

## 3. Results and discussion   

### 3.1. MT quantification   

MT quantification measurements were carried out using the low-resolution mode, with a 2 × 2 mm beam size and the detector widely opened, in order to integrate the maximum of scattered signal of MT broadened signal. The pole figure method is employed mainly for graphic visualization of the relative MT content. The absolute quantification of MT fractional volume of the GaP layer is then carried out by the rocking-curve method.

#### 3.1.1. Visualization by pole figure method   

**Figure 1:**
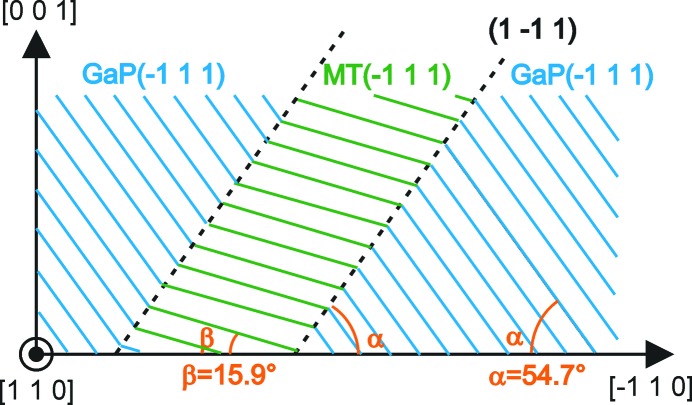
Geometrical sketch of nominal GaP and MT plane inclination in a thin film of GaP. The nominal GaP {111} planes are inclined by 54.7° from the (001) plane, while MT formation creates additional (111)-type planes inclined by 15.9°.

Fig. 1 ▶ represents a geometrical sketch of the MT plane inclination in a thin film of GaP. The nominal GaP {111} planes are inclined by 54.7° from the (001) plane, while MT formation creates additional {111}-type planes inclined by 15.9°. The principle of the XRD pole figure method is to fix the 2θ and ω angles to the GaP 111 Bragg position and to observe these (111)-type planes by bringing them perpendicular to the scattering vector. This entails ϕ scans with successive χ values, where ϕ corresponds to the sample rotation around its surface normal and χ the inclination angle of the sample surface with respect to the scattering plane.

**Figure 2:**
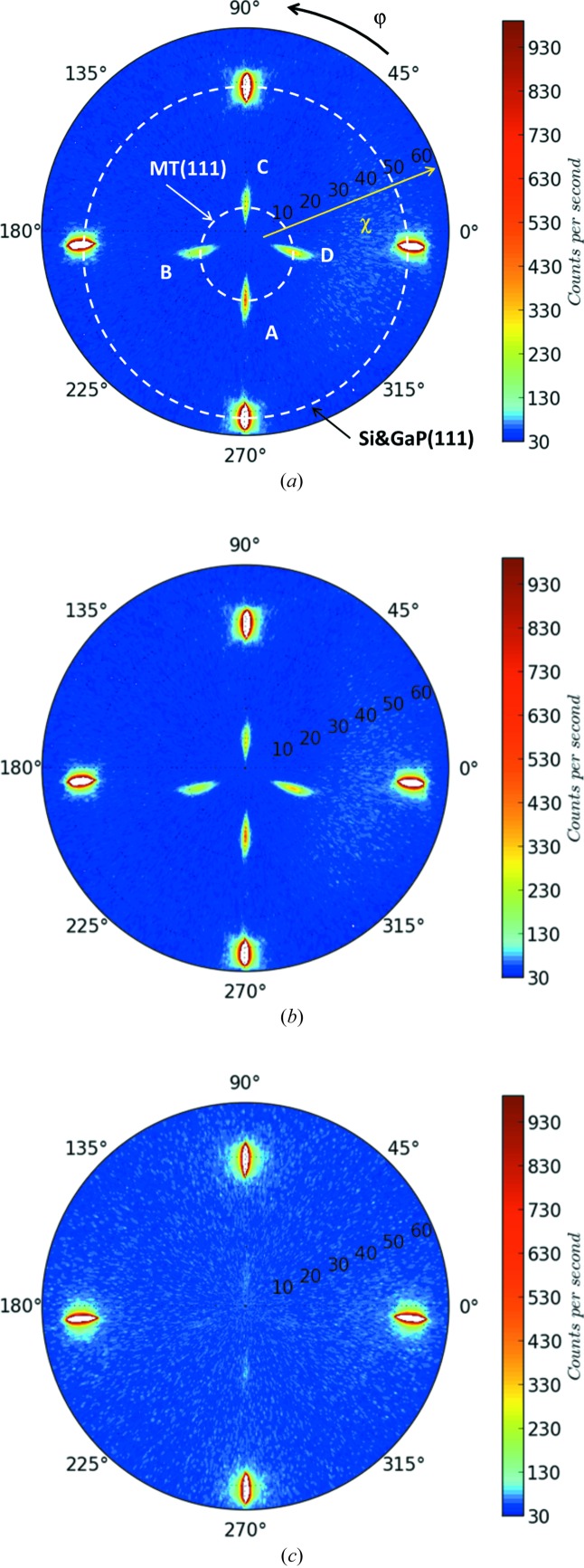
XRD poles figures showing the MT signals of the 45 nm GaP/Si samples: (*a*) S1, (*b*) S2 and (*c*) S3. The angle and the distance in the polar coordinate system correspond, respectively, to ϕ and χ. The four spots between χ = 10 and 20° represent the MT signals, and the other four between 50 and 60° are due to Si and GaP 111 reflections.

Fig. 2 ▶(*a*) shows a typical pole figure taken for S1. Owing to the fourfold symmetry of the zinc-blende GaP crystalline structure, four MT variants are generally observed on the pole figure around χ = 16°, with ϕ = 0, 90, 180 and 270°. We note the MTs elongated in real space parallel to the atomic step boundaries MT-A and MT-C, and those perpendicular to the step boundaries MT-B and MT-D, in agreement with the notation of another research group (Skibitzki *et al.*, 2012 ▶). Notice that Skibitzki and co-workers used a goniometer head to maintain the Si [001] nominal direction parallel to the ϕ rotation axis. Despite the miscut, their pole figure was well centred. This requires, however, the alignment of two supplementary angles. In our case no goniometer head can be added to the setup. Therefore, MT-A and MT-C are shifted in χ by ±6°, as well as the corresponding 111 nominal reflections. This is due to the substrate miscut. For the two other azimuths, a ϕ shift and MT reflection distortion are observed. Besides, the four spots near χ = 55° correspond to GaP 111 nominal reflections. In reality, the low-resolution XRD does not allow the GaP and Si reflections to be separated, and thus the diffracted intensity shown in the figure comes mainly from the Si substrate because of its far higher effective scattering volume as compared with the GaP thin layer.

The pole figures of samples S2 and S3 are represented, respectively, in Figs. 2 ▶(*b*) and 2 ▶(*c*). The diffracted intensities have been normalized with that of the incident beam and are presented on the same intensity scale for comparison. Assuming that the integrated intensities of the MT spots are directly proportional to the MT volume inside the GaP layer, the reduction of MT reflection intensity of S2 and especially S3 in comparison with S1 indicates MT elimination during the evolution of sample growth conditions. This is in agreement with absolute quantification results obtained by the rocking-curve method that will be introduced later.

The pole figure method is a very simple and illustrative way of MT characterization since on the one hand it is very simple to carry out, requiring only a rough alignment of the *z* position (the *z* direction is perpendicular to the sample surface), and on the other hand the visualization of MTs gives rapid feedback for comparison of the samples and optimization of the growth conditions.

#### 3.1.2. Absolute quantification by the rocking-curve method   

In order to evaluate the MT volume fraction, four rocking-curve (RC) scans were performed, respectively, on the four MT variants, with 2θ fixed at the GaP 111 Bragg position (*i.e.* 2θ_B_ = 28.44°) and the sample rotated on the ω axis around its Bragg position ω_B_ (ω_B_ = θ_B_ without miscut). The 6° substrate miscut is then taken into account by the ω shift for MT-B and MT-D (*i.e.* ω = θ_B_ ± 6° with χ = 16°), and by the χ shift for MT-A and MT-C (*i.e.* χ = 16 ± 6° with ω = θ_B_). In addition, one RC scan around the nominal GaP 002 reflection is carried out, to measure the volume fraction of the nominal GaP phase. The experimental integrated intensity (in counts) diffracted by MTs or the nominal phase from the RC is simply measured from
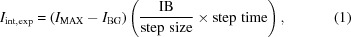
where *I*_MAX_ is the maximum measured intensity, *I*_BG_ is the average background intensity, IB is the integral breadth of the profile, step size is the scan increment and step time is the acquisition time for each scan step.

Next, the theoretical integrated intensity diffracted by a small single crystal rotated around the Bragg position can be calculated by the following equation, when absorption is neglected, as explained in the book by B. E. Warren (1990 ▶):
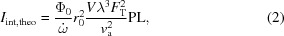
where Φ_0_ is the intensity of the incident beam in counts/(seconds × unit area), 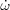 is the constant angular velocity of the crystal rotation, *r*_0_ is the classical radius of the electron and 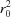 the scattering cross section of the electron, *V* is the volume of the crystal, λ is the incident X-ray wavelength, *v*_a_ is the crystal unit-cell volume, 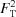 is the unit-cell structure factor taking into account the Debye–Waller factor, and PL is the Lorentz–polarization factor.

**Figure 3:**
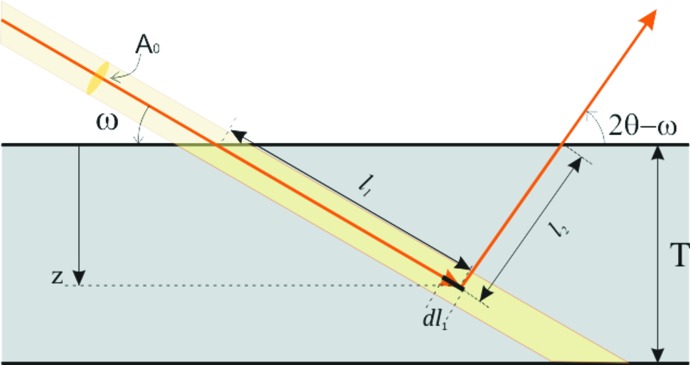
Sketch of effective scattering volume in a GaP thin layer, with *I*_0_ and *A*_0_ the intensity and cross section of the incident X-rays, ω and 2θ − ω the incident and emergent angles, and *T* the layer thickness.

We will consider the real case of an X-ray beam scattered by a GaP monocrystaline thin-layer sample, as shown in Fig. 3 ▶. We define *I*_0_ and *A*_0_ as the intensity and cross section of the incident X-ray, ω and 2θ − ω as the incident and emergent angle, and *T* as the thickness of the GaP layer. The fact that the flux density reduces owing to absorption before and after elastic Bragg scattering gives rise to the effective scattering volume, *i.e. V*.

According to the Beer–Lambert law, the intensity diffracted by the elementary crystal volume d*V* = d*l*_1_ *A*_0_ located at a depth of *z* can be expressed by the following equation:
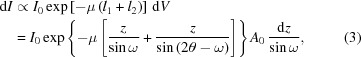
where *l*_1_ and *l*_2_ are the lengths of the X-ray path and μ is the linear attenuation coefficient of the material. The integration of the equation on d*z* through *T* permits us to obtain the effective scattering volume of the crystal, *V*:
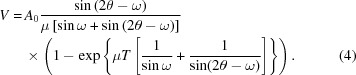
In our study, the beam could be considered as unpolarized without the use of a monochromator. The Lorentz–polarization factor is assumed to be 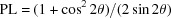. Warren (1990 ▶) has detailed the calculation of *F*_T_, and we have applied the approximation of Kushwaha (1987 ▶) for the Debye–Waller factor.

The final equation becomes
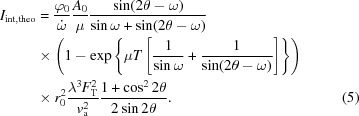
Finally, the 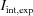/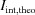 ratio is used to estimate the volume fraction of the nominal GaP phase and each MT variant. Table 1 ▶ lists for the three 45 nm GaP/Si samples the volume fractions of the four MT variants (*i.e.* MT-A, MT-B, MT-C and MT-D), the sum of all MT variants (MT-S), the nominal phase (NP), and the total of the nominal phase and MTs. A significant anisotropy is observed among the different MT variants, as already noticed (Skibitzki *et al.*, 2012 ▶; Quinci *et al.*, 2013 ▶), and MT-A systematically corresponds to a larger volume fraction, which is also obvious from the pole figures. This suggests an influence of the atomic step on MT generation. A first reduction of MT volume fraction from 24.6 to 14.8% is observed while using Ga as prelayer instead of P. A more drastic reduction of MT volume fraction has been achieved for sample S3.

By summing the different volume fractions, it can be noticed first that these measurements lead to a total near the expected 100%, varying from 76.9 ± 8.4% to 111.6 ± 13.1%. This sum is less than 100% for samples of higher MT density (S1 and S2), while it is greater than 100% for S3. Several phenomena may lead to such disagreements. The presence of very small intercrossing MTs (especially in the case of high MT density), which are difficult to integrate correctly with a limited area point detector, leads to an underestimation of the MT volume fraction. The presence of APDs, producing a broadening of the GaP 002 reflection that is only partially integrated (along the [110] direction in this case), leads to an underestimation of the nominal phase volume fraction. These factors contribute to the sum being lower than 100%. Contrarily, atoms located at MT and nominal phase boundaries contribute both to the GaP 002 reflection and to the 111 reflections of the MT variant. These atoms are counted twice, which may yield a total volume fraction higher than 100%. But, a more systematic study should be carried out to evaluate the sources of disagreement.

**Figure 4:**
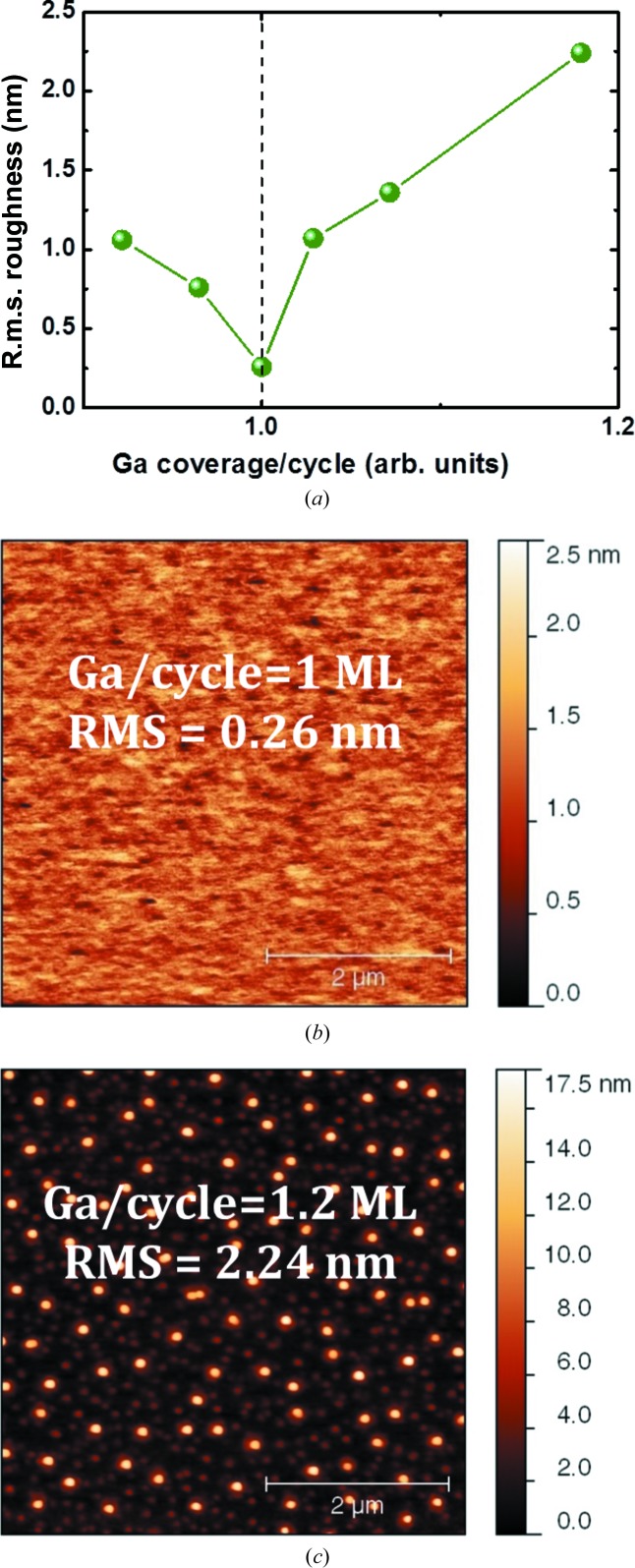
5 × 5 µm AFM images of the 20 ML GaP/Si sample grown by MEE with (*a*) the AFM r.m.s. roughness *versus* Ga coverage graph: (*b*) 1 ML Ga per cycle and (*c*) 1.2 ML per cycle. The dashed line is a guide for the eyes.

Two important results arise from the laboratory setup XRD analysis. First, the quantification reveals a high degree of MT volume fraction for MBE-grown samples, with a slight advantage for S2 (Ga prelayer), in agreement with the study by Bi *et al.* (1996 ▶), who claimed better results with respect to a P prelayer. Second, as compared to S1 and S2, S3 exhibits a drastic improvement of the structural quality, with a total MT volume fraction of about 1.3%. This result has been obtained after a long optimization study. First of all, the MEE technique was employed because the alternated growth of Ga and P allows a high degree of control of Ga content and incorporation. Secondly, GaP grown at high temperature (above 693 K) has been observed by Narayanan *et al.* (1999 ▶) to exhibit low MT content, as predicted by Ernst & Pirouz (1988 ▶), but our previous study showed a higher surface roughness detrimental for optoelectronic device applications, which lead to an optimization of the MEE growth procedure at 623 K. Then we employed the two-step procedure consisting of the MEE technique followed by conventional MBE, as suggested by Grassman *et al.* (2009 ▶), to enhance the two-dimensional formation by MEE at low temperature and to eliminate the MT generation by MBE at higher temperature. Finally, slight differences of Ga prelayer coverage during the first stage of MEE growth were found to play an important role. Fig. 4 ▶ presents AFM measurements of the surface topography after growth of 20 monolayers of MEE GaP at 623 K for different Ga amounts per MEE cycle. The r.m.s. roughness measured each time from AFM images indicates a minimal roughness considered to correspond to one monolayer (ML) of Ga coverage, as shown in Figs. 4 ▶(*a*) and 4 ▶(*b*). This study also showed that, beyond the roughness criterion, a slight Ga coverage excess causes Ga droplets and is detrimental for surface quality, as shown in Fig. 4 ▶(*c*). Therefore, a 0.9 ML Ga/MEE cycle was chosen for the growth of the 45 nm-thick GaP sample S3. Though this low MT content is hardly detectable using a conventional laboratory setup, it still represents too high a density of planar defects, mostly threading to the surface, as shown hereafter by APD quantification carried out on S4, grown under the same conditions as S3 for its first 10 nm GaP layer.

### 3.2. Antiphase domain quantification   

#### 3.2.1. High-resolution setup XRD   

Quantification of defects using RSMs around the GaP 002, 004 and 006 nearly specular reflections has been demonstrated in our previous work (Létoublon *et al.*, 2011 ▶; Guo *et al.*, 2012 ▶; Nguyen Thanh *et al.*, 2012 ▶). The experiment was performed in high-resolution mode with the detector in PSD mode to ensure a rapid characterization and a good separation of the GaP and Si signals. The APD being considered as a simple exchange of Ga and P positions compared to the main phase, the scattered intensity (*I*) around a Bragg reflection is given by the following equations:
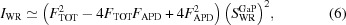


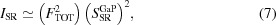
where the subscripts WR and SR represent, respectively, weak reflections where *h* + *k* + *l* = 2*n* + 2 and strong reflections where *h* + *k* + *l* = 4*n*, with *n* an integer; *F*_APD_ and *F*_TOT_ are the form factor of APD and that of the whole thin layer; 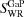 and 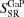 are the unit-cell structure factors of the GaP main phase for weak reflections and strong reflections, respectively. From the two equations, we can find that, for the weak reflections like 002 and 006, the APD contribution to the scattered intensity is enhanced by a factor of 4 (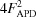) and the long-range lateral coherence of the layer remains with the term 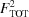. The presence of APD has theoretically no influence on the strong reflections like 004, however. The transverse scans along the lateral direction display two-component line shapes composed of a resolution-limited thin peak, implying a long-range structural correlation length, and a diffuse-scattering broad component, implying a shorter-range correlation length, as already widely investigated in the mosaic epitaxial GaP thin films (Takagi *et al.*, 2010 ▶; Durand *et al.*, 2011*a* ▶). The integral breadth (IB) of the thin peak indicates a very regular lattice spacing and good parallelism of the epitaxial GaP atomic planes, over a relatively long distance. The broad peak around the 002 or 006 reflection has been mainly attributed to APDs, and the broadening of 004 is considered to originate mainly from other planar defects like MTs or stacking faults.

**Figure 5:**
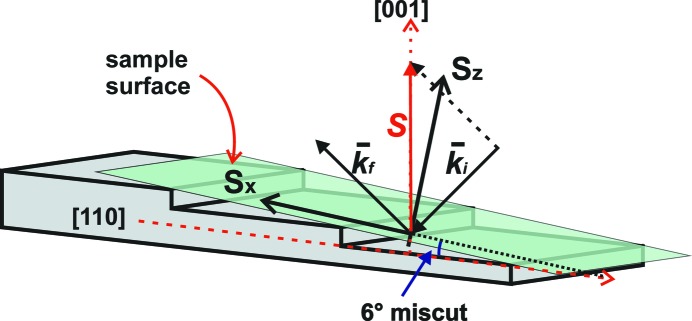
Illustration of the scattering geometry of Si(001) substrates misoriented by 6° toward the [110] direction.

The RSMs around the 002, 004 and 006 reflections were obtained for extraction of lateral transverse scans in order to study the lateral correlation lengths of the planar defects. Fig. 5 ▶ illustrates the X-ray scattering geometry for reciprocal space mapping in the system of Si(001) substrates misoriented by 6° toward the [110] direction. 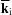 and 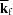 are the reduced scattering vectors along, respectively, the incident and scattered directions, with |
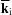
| = |
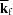
| = 1/λ. The vector **S** is defined as 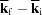 and is collinear with [001]. The in-plane direction (*S* *_x_*) is coplanar with the scattering plane defined by 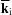 and 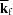 and parallel to the projection of **S** ([001] vector) onto the sample surface. The out-of-plane direction (*S*_*z*_) is the growth direction that is normal to the surface. For this azimuthal scattering condition, 00l reflections are not strictly specular since the 6° miscut results in a shift of ω (*i.e.* ω = θ + 6°), which also allows the highest transverse resolution (when the miscut results in a nonzero value of the χ angle, a significant broadening of transverse scans is indeed observed owing to a rotation of the linear beam footprint). The advantage of using such (*S*_*x*_, *S*_*z*_) coordinates in this azimuthal condition is that the high perfection of a thin epitaxial layer (sharp interface, flat surface, low plastic relaxation, low defect density…) results in an elongation of the epilayer reciprocal lattice node (RLN) along *S*_*z*_ with thickness fringes that fall at the same *S* *_x_* positions as the substrate RLN. The extraction of ‘transverse scans’ along *S*_*x*_ also allows measurements of the correlation length in the in-plane direction with a high sensitivity, by summing a certain height along *S*_*z*_ (as depicted on the RSM) without resolution loss.

**Figure 6:**
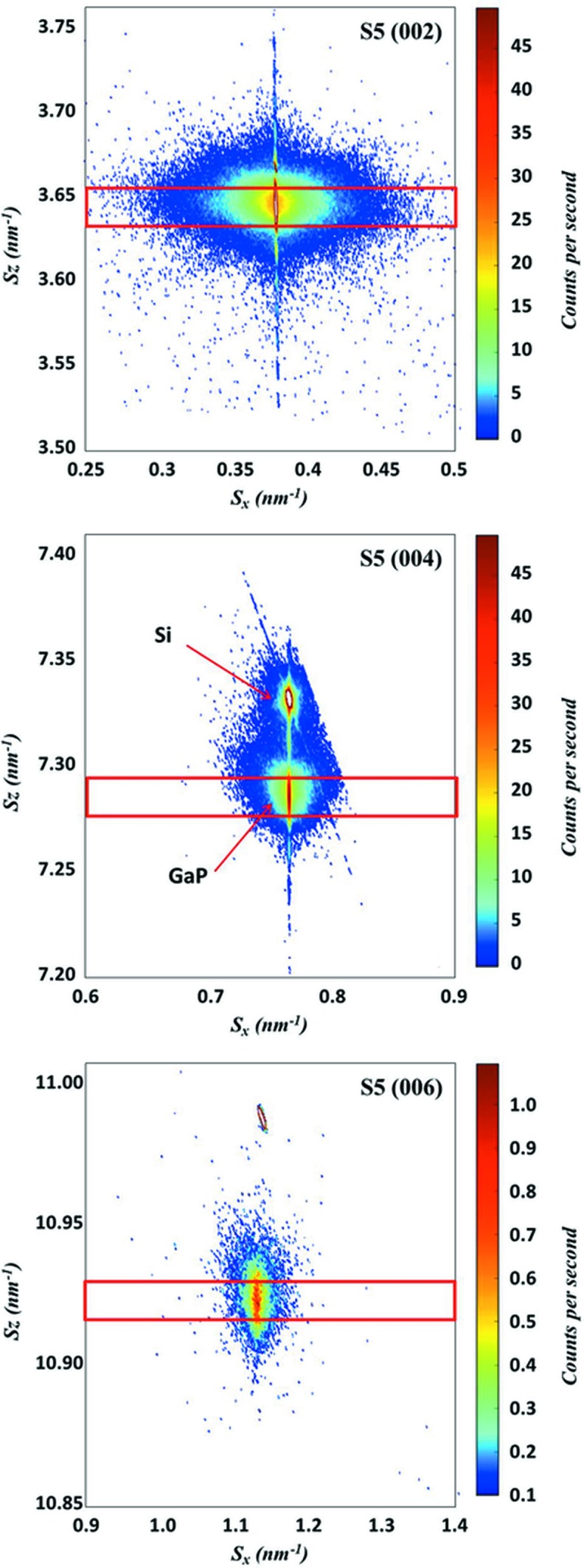
XRD RSM around 002, 004 and 006 nearly specular reflections for S5. The red line rectangles indicate the *S_z_* boundaries for transverse scan extraction.

**Figure 7:**
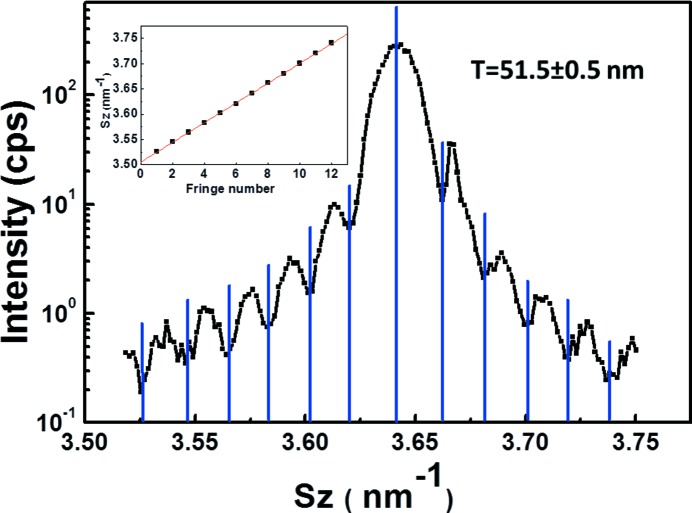
*S_z_* profile at the central *S_x_* position of the 002 RSM for S5. The inset represents the measurement of layer thickness from the fringes.

S4 and S5 were selected for a thorough analysis after demonstration of their high structural quality with low plastic relaxation and low MT volume fraction (nearly undetectable for S4 and 1.5 ± 1.5% measured form RC scans for S5). Fig. 6 ▶ shows the RSMs of S5 around the 002, 004 and 006 nearly specular reflections. The RLN broadening of GaP along *S*_*z*_ is mainly due to the layer thickness, which causes the so-called crystal truncation rod (CTR). A more diffuse broadening observed on weak reflections is believed to correspond mainly to threading defects. The *S*_*z*_ profile on the 002 reflection is extracted at the central *S*_*x*_ position and shows clear and regular thickness fringes, as shown in Fig. 7 ▶, indicating good structural quality of the thin epitaxial layer (low defect density, and low surface and interface roughness). The inset represents the measurement of layer thickness from the fringes, and the thickness turns out to be 51.5 ± 0.5 nm, which confirms the nominal layer thickness of 45 nm. The 004 RSM exhibits both GaP and Si peaks (Si is a forbidden reflection and generally very weak on 002 and 006), with the GaP peak lying at the same *S_x_* value as Si, owing to the absence of plastic relaxation. Intensity integration was performed at the centre of the GaP CTR along *S_x_* to extract the transverse scans. The same measurements were carried out for S4.

The transverse scan profiles are fitted by two pseudo-voigt functions (Young & Wiles, 1982 ▶), respectively, for the resolution-limited thin component and the broad component. Table 2 ▶ shows the integral breadth of the broad peak for each reflection, as well as the quality factor (QF) for 002. The QF is defined as the area ratio of the thin peak to the broad peak. A higher QF indicates better crystalline quality without many planar defects along the lateral direction (*S_x_*). Hence the first analysis on QF indicates a higher crystalline quality for S5.

A more precise evaluation has been carried out based on the Williamson–Hall evaluation method (Williamson & Hall, 1953 ▶; Herres *et al.*, 1996 ▶; Kirste *et al.*, 2005 ▶), as described by Durand *et al.* (2011*b* ▶). Herres and co-workers attributed the line profile broadening to three mechanisms: the tilt, the average crystallite size and the inhomogeneous strain. However, in our case, broadening due to inhomogeneous strain along *S_z_* does not affect the 00l *S_x_* transverse scans (Miceli & Palmstrom, 1995 ▶). Therefore, we take into account only the mosaicity tilt of the crystallites (Δ*M*) relative to the sample surface and the lateral correlation length (ξ*_x_*) corresponding to the mean size of the defects.

The IB of a Voigt function (β) is calculated using the following relationship (Halder & Wagner, 1966 ▶), taking into consideration both the IB of the Lorentzien component (β_L_) and that of the Gaussian component (β_G_): 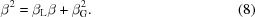
The IB due to the lateral correlation length is considered to be β_*x*_ = 1/ξ*_x_* according to Scherrer’s law and can be modelled by a Lorentzien function. The IB due to the mosaicity tilt, denoted β_Δ*M*_, is given by β_Δ*M*_ = Δ*MS*, with *S* the scattering vector modulus, and can be modelled by a Gaussian shape. By applying these conditions to equation (8), we obtain the following relationship, where β(*S*) is the measured broad peak IB of transverse scans for different diffraction order reflections (*i.e.* 002, 004 or 006): 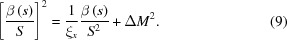
Then, if we plot 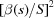 as a function of 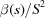, all reflection points should form a straight line in the ideal case. Δ*M* and ξ*_x_* are, respectively, given by the intercept and the slope. We call this a ‘Williamson–Hall-like’ (WHL) plot (Williamson & Hall, 1953 ▶). Here, because the 002 and 006 transverse scan broadening is considered to be mainly due to APDs, as explained above, Δ*M* and ξ_*x*-APD_ are extracted from these two points only. The extracted ξ_*x*-APD_ corresponds to the average APD size in the corresponding scan direction in the case of a low density of defects of another nature. It also corresponds to the distance between two APBs, in the case of high APB density (Létoublon *et al.*, 2011 ▶) and equilibrium between phase and antiphase domains. This correspondence has been already confirmed by several observations carried out on the same samples by TEM/STEM and XRD. Moreover, since the Δ*M* in the crystal remains the same for the 004 RSM, the straight line connecting the 004 point and the intercept permits the extraction of the correlation length related to other types of planar defects (ξ_*x*_).

**Figure 8:**
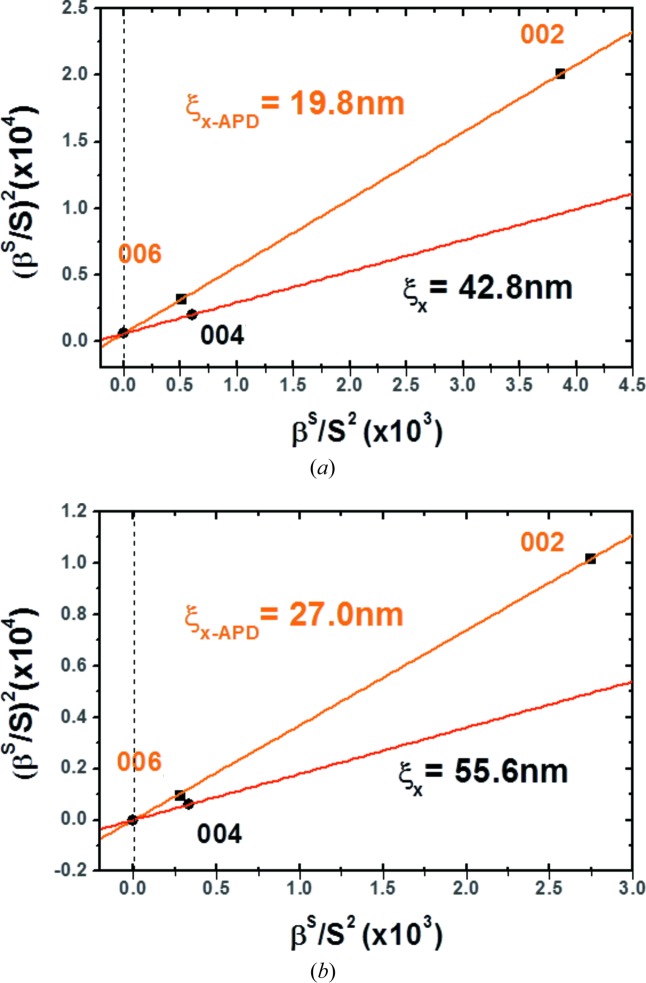
WHL plots using the 002, 004 and 006 reflections for (*a*) S4 and (*b*) S5. The intercept corresponds to the mosaicity tilt, and the defect correlation length is deduced from the corresponding slope.

Fig. 8 ▶ presents the WHL plots for both S4 and S5. The ξ_*x*-APD_ for S5 is measured to be 27.0 ± 1.8 nm, greater than that for S4 (19.8 ± 1.2 nm) and the previous samples (Guo *et al.*, 2012 ▶), but still gives an APB density much too high for optoelectronic device applications. The ξ_*x*_ values related to other defects for S4 and S5 are, respectively, 42.8 ± 2.5 and 55.6 ± 3.3 nm, which also confirms a better crystalline quality for S5 with smaller defect density.

#### 3.2.2. Cross-section STEM-BF analysis   

**Figure 9:**
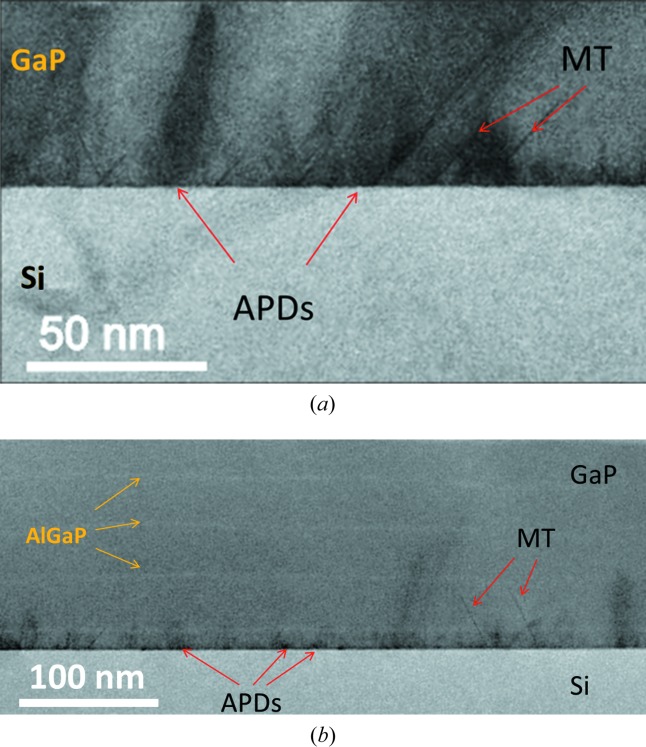
Cross-section STEM-BF images for (*a*) S5 and (*b*) S6.

Fig. 9 ▶ shows cross-section STEM-BF images for samples S5 and S6 (for which no MT signal is detected using the above-presented XRD analytical methods). The APDs of sample S5 with boundaries lying on nearly (110)-oriented planes are shown in Fig. 9 ▶(*a*). Most boundaries are threading to the surface. The mean APB distance is of the order of 30 nm. These observations are in very good agreement with the XRD analysis. This confirms the pertinence and reliability of our XRD method, which has the strong advantages of being a nondestructive method without sample preparation and with statistical averaging over a large area. This image also shows the presence of very thin MTs that could not be detected by using either the rocking-curve or the pole figure method on our XRD setup. The last presented sample served as a test for the annihilation process of APDs, with a first 10 nm-thin MEE layer followed by MBE growth of GaP with AlGaP marker layers. Most APDs are shown to be annihilated within the first 10 nm, and a larger-field observation showed a progressive annihilation through the layers with a final density of about 3 APBs per micrometre at the top (Fig. 9 ▶ *b*).

For lower defect density generation, GaP can be grown on top of a double Si step surface, optimal for APD annihilation. Such an Si surface has already been obtained in our laboratory and was grown in a UHV-CVD chamber connected to the III–V MBE chamber (Quinci *et al.*, 2013 ▶).

## 4. Conclusion   

Relative MT content has been visualized using the pole figure method. Quantification of MT volume fraction has been performed on rocking-curve scans for different GaP thin layers lattice matched on Si substrates and showed results consistent with the measured volume fraction of the nominal phase. These methods have been applied for optimization of the growth procedure and allowed us to obtain a dramatic reduction of the MT volume fraction. A thorough analysis carried out on high-structural-quality samples showed evidence of lateral broadening of the specular weak reflections that has been correlated to antiphase domains. The correlation length determined from integral breadths from the two weak reflections gave the APB mean distance. This value is consistent with cross-section STEM observations.

A final optimized sample grown for STEM-BF observation showed very promising results, with very low MT density and self-annihilation of most APDs. This opens the route for the realization of optoelectronic devices monolithically integrated on silicon.

## Figures and Tables

**Table 1:** Volume fractions (%) of MTs and the nominal phase measured from the rocking-curve scans for the series of 45nm GaP/Si samples

Sample	MT-A	MT-B	MT-C	MT-D	MT-S	NP	NP+MT-S
S1	7.8	6.4	5.2	5.3	24.6 2.5	69.2 7.0	93.8 9.5
S2	4.6	3.4	3.0	3.8	14.8 1.6	62.1 6.8	76.9 8.4
S3	0.4	0.4	0.3	0.2	1.3 1.0	110.3 12.1	111.6 13.1

**Table 2:** Integral breadths of diffuse-scattering components extracted from the transverse scans of the 002, 004 and 006 reflections on samples S4 and S5, and quality factor calculated from the 002 reflection

	002		004	006
	IB (nm^1^)	QF	IB (nm^1^)	IB (nm^1^)
S4	0.052	0.21	0.033	0.062
S5	0.037	0.98	0.018	0.034
